# *Hypoxis hemerocallidea* Significantly Reduced Hyperglycaemia and Hyperglycaemic-Induced Oxidative Stress in the Liver and Kidney Tissues of Streptozotocin-Induced Diabetic Male Wistar Rats

**DOI:** 10.1155/2016/8934362

**Published:** 2016-06-15

**Authors:** Oluwafemi O. Oguntibeju, Samantha Meyer, Yapo G. Aboua, Mediline Goboza

**Affiliations:** ^1^Nutrition & Chronic Disease Research Group, Oxidative Stress Research Centre, Department of Biomedical Sciences, Faculty of Health and Wellness Sciences, Cape Peninsula University of Technology, P.O. Box 1906, Bellville 7535, South Africa; ^2^Department of Biomedical Sciences, Faculty of Health and Wellness Sciences, Cape Peninsula University of Technology, P.O. Box 1906, Bellville 7535, South Africa; ^3^Faculty of Health and Wellness Sciences, Cape Peninsula University of Technology, P.O. Box 1906, Bellville 7535, South Africa

## Abstract

*Background*.* Hypoxis hemerocallidea* is a native plant that grows in the Southern African regions and is well known for its beneficial medicinal effects in the treatment of diabetes, cancer, and high blood pressure.* Aim*. This study evaluated the effects of* Hypoxis hemerocallidea* on oxidative stress biomarkers, hepatic injury, and other selected biomarkers in the liver and kidneys of healthy nondiabetic and streptozotocin- (STZ-) induced diabetic male Wistar rats.* Materials and Methods*. Rats were injected intraperitoneally with 50 mg/kg of STZ to induce diabetes. The plant extract-*Hypoxis hemerocallidea* (200 mg/kg or 800 mg/kg) aqueous solution was administered (daily) orally for 6 weeks. Antioxidant activities were analysed using a Multiskan Spectrum plate reader while other serum biomarkers were measured using the RANDOX chemistry analyser.* Results*. Both dosages (200 mg/kg and 800 mg/kg) of* Hypoxis hemerocallidea* significantly reduced the blood glucose levels in STZ-induced diabetic groups. Activities of liver enzymes were increased in the diabetic control and in the diabetic group treated with 800 mg/kg, whereas the 200 mg/kg dosage ameliorated hepatic injury. In the hepatic tissue, the oxygen radical absorbance capacity (ORAC), ferric reducing antioxidant power (FRAP), catalase, and total glutathione were reduced in the diabetic control group. However treatment with both doses improved the antioxidant status. The FRAP and the catalase activities in the kidney were elevated in the STZ-induced diabetic group treated with 800 mg/kg of the extract possibly due to compensatory responses.* Conclusion*.* Hypoxis hemerocallidea* demonstrated antihyperglycemic and antioxidant effects especially in the liver tissue.

## 1. Introduction

Diabetes mellitus (DM) is becoming one of the leading causes of death worldwide because of its adverse complications that include cardiovascular related diseases and chronic kidney disease [1–3]. Proper management and treatment of diabetes mellitus are lacking, especially in developing countries, which possibly explains the escalating percentages of morbidity and mortality of the disease in developing countries particularly in Africa [4, 5]. It is projected that DM will become the 7th leading cause of mortality by the year 2030 [6]. It has also been predicted that 552 million people will be diabetics by 2030 and currently; 382 million people are diabetics [7, 8]. The rate of incidence of DM is so alarming that reports projected that 1 in 10 nondiabetic adults will become a diabetic patient by the year 2030 [6]. Because of the limitations associated with the present antidiabetic agents, it is therefore important to explore alternative and/or complementary agents/remedies in the treatment and management of DM.

The hyperglycemic state in diabetes has been attributed as the major factor that triggers the development of both acute and long term changes in the cellular metabolism of different molecules [9]. Altered metabolism of macromolecules ultimately triggers excessive formation of free radicals via different pathways as a result speeding up the development of diabetic complications [10–12]. Diabetes mellitus has been associated with hepatic and renal injuries where the prevalence of diabetic hepatic disease in diabetic patients has been estimated to lie between 17 and 100% and 20 and 40% for diabetic kidney disease (DKD) [13, 14]. Diabetic hepatic disease is marked by elevated serum hepatic enzyme activities as a consequence of ROS [15]. The functional changes in DKD that accompany structural changes in the kidney include increased tubule-glomerular feedback, increase in systemic blood pressure, decreased creatinine clearance and glomerular filtration rate, proteinuria, and glomerular hyperperfusion [16]. Oxidative stress still remains the primary factor that drives the development of both functional and structural changes in diabetic kidney disease [15].

Hypoxis is a family of plants that are extensively used for medicinal purposes in the Southern African region. This family consists of several types of species which include* H. interjecta*,* H. multiceps*,* H. nyasica*,* H. obtuse*,* H. sobolifera*, and the* H. hemerocallidea*. The hypoxidaceae super family is made up of 8 genera and 130 species [17].

Taxonomically,* Hypoxis hemerocallidea* belongs to the hypoxidaceae (Star lily family). This plant was first described by Linnaeus in 1759. The name was derived from the Greek words* hypo* (below) and* oxy* (meaning sharp), with reference to the ovary which is pointed at the base. The plant has recently drawn attention of researchers because of its beneficial medicinal effects. Geographically, the plant is mostly distributed in the southern hemisphere and is mostly abundant in Southern Africa [18].

*H. hemerocallidea* is commercially known as the African potato and has been also referred to as the miracle plant [17]. The plant is characterised by strap shaped leaves held on thick green hairy stems that are unbranched and the stems hold stalks supporting 2–12 yellow, star shaped flowers [3, 19].

**Figure 1:**
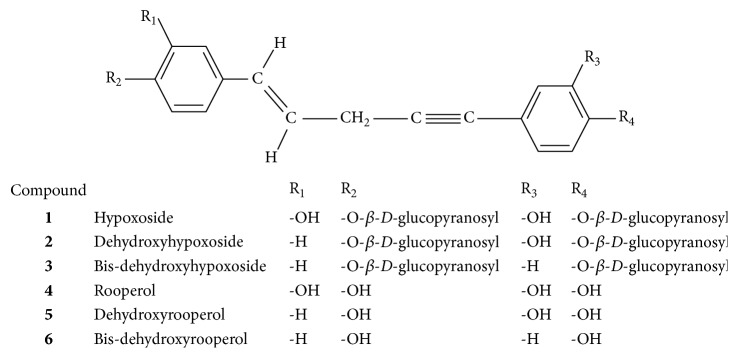
Chemical structures of norlignans derived from hypoxide (**1**–**6**) (adapted from Laporta et al. [22]).

The tuberous part of the* H. hemerocallidea* is the one that is believed to possess bioactive compounds [20]. In folk medicine, the African potato had been used for centuries to treat a catalogue of ailments that includes the following: arthritis, diabetes mellitus, high blood pressure, and cancer [21]. The therapeutic effects of the African potato were attributed to the presence of sterols, sterols, sterolins, norlignan, daucosterol, and rooperol [3, 17–21]. The chemical structure of norlignan derived from hypoxide is shown in Figure 1. Among these phytochemicals, daucosterol, beta sitosterol, and rooperol are mostly associated with therapeutic activities [21]. Rooperol a dicatechol aglycone bioactive compound which has been extensively studied has been reported to exhibit powerful antioxidant, anti-inflammatory, and immune properties in human blood [20, 22]. Hypoxide is the major component isolated from the corm. Hypoxide ((E)-1,5 bis-(4′-*β* D-glucopyranosyloxy-3′-hydroxyphenyl)pent-4-en-1-yne) is a glycosylated norlignan that is derived from cinnamic acid [22]. In its natural form, hypoxide is inactive but can be hydrolysed to rooperol by the action of *β* glucosidase enzyme. This conversion occurs in the gastrointestinal system particularly in the large intestines in humans and in animals, and bacterial *β* glucosidase catalyses the conversion.

This study investigated the effects of* Hypoxis hemerocallidea* in the kidney and liver tissue of STZ-induced diabetic male Wistar rats by assessing the antioxidant activities and selected biomarkers. To the best of our knowledge, this is the first time a study would focus mainly on the antioxidant activities as well as their potential deleterious effects using animal model.

## 2. Materials and Methods

### 2.1. Animals

The ethical approval was obtained from the Research Ethics Committee (REC), Faculty of Health and Wellness Sciences of the Cape Peninsula University of Technology (ethical certificate reference number NHREC:REC-230408-014). All animals used received humane care from trained personnel and were treated with respect according to the principles of Laboratory Animal Care of the National Society of Medical Research and the National Institutes of Health Guide for the Care and Use of Laboratory Animals of the National Academy of Sciences [23]. The animals were purchased from University of Stellenbosch, Department of Physiological Sciences (South Africa). Animals were housed under standard environmental conditions (23 ± 1°C, 55 ± 5% humidity) and were exposed to free water access, standard animal diet called standard rat chow (SRC), and* Hypoxis hemerocallidea* aqueous extract depending on the group. Each group of animals (12 animals per group) received the same amount of water and SRC daily. Animals were housed in stainless steel cages with surfaces made of movable plastic for easy daily cleaning and maintenance. The animal feeding was conducted over a period of 6 weeks.

### 2.2. Induction of Diabetes

Diabetes was induced in overnight fasted experimental groups via single intraperitoneal injection of streptozotocin (STZ) at a dose of 50 mg/kg body weight. The STZ was dissolved in 0.1 M cold sodium citrate buffer, pH 4.5. After 72 hrs, fasting blood glucose levels of all the animals were measured from blood obtained from the rat tail vein. Experimental animals with blood glucose levels greater than 15 mmol/L were considered to be diabetic using the Accucheck Glucometer (Roche, Germany).

### 2.3. Plant Materials

Powdered methanolic extract of the* Hypoxis hemerocallidea* corm was obtained from Afriplex, kept in an aluminium foil sealable bag and stored in a −90 degrees Celsius refrigerator. The identification and authentication of the plant were done by Botanist at Afriplex, Parrow, Cape Town, South Africa, with voucher number (CRMD02093) and kept at the herbarium. The extraction of the plant extract was done by Afriplex (the company that commercially produces it for therapeutic purpose).

### 2.4. Study Design

Rats weighing between 210 and 240 g were divided randomly into five groups: group 1: normal control; group 2: diabetic control; group 3: STZ + 800 mg/kg* Hypoxis hemerocallidea*; group 4: STZ + 200 mg/kg* Hypoxis hemerocallidea*; group 5: normal + 800 mg/kg* Hypoxis hemerocallidea*. Each group consist of twelve animals (*n* = 12). Body weights were measured whenever glucose readings were taken. Different concentrations of the extracts were made up in 1 mL of distilled water daily (200 mg/kg and 800 mg/kg body weight). Experimental and control groups were gauged with designated concentrations of the plant extract and distilled water, respectively. Treatment of rats with aqueous plant extract was done daily from the fourth day to the second last day of the 6-week period.

### 2.5. Blood and Tissue Collection

After the treatment period, final fasting blood glucose levels and body weights were recorded prior to sample collection. Rats were anaesthetised by the use of 1 mL of 100 mg/kg of sodium pentobarbitone via the intraperitoneal injection. Blood samples were collected from the aorta using 5 mL syringes connected to 25-gauge hypodermic needles into 10 mL serum separator vacutainer tubes (yellow top tubes). Tubes were allowed to stand at room temperature for 5–10 minutes before being centrifuged at 3500 rpm for 15 minutes. Once centrifuged, the serum was transferred into cryotubes, frozen in liquid nitrogen and stored at −80 degrees Celsius.

Tissue samples were collected on ice, weighed, washed in phosphate buffer saline (PBS) to remove blood, and blotted to remove excess PBS. The left side of the livers was minced into small pieces that were transferred into cryotubes, frozen in the liquid nitrogen, and finally stored at −80 degrees Celsius for future analysis. The same procedure was also applied to the kidney samples. Stored tissue samples were homogenised in PBS mixed with 0.5% v/v% triton X-100 at pH; 7.5. 200 mg of tissue was added to 2000 *μ*L PBS and homogenised on ice. Homogenates were centrifuged at 4 degrees Celsius at a speed of 14000 rpm for 10 minutes. The supernatants were then transferred into labelled tubes and stored at −80°C.

### 2.6. Determination of Relative Tissue Weights

Relative tissue weights were estimated by comparing the tissue weight to the total body weight (relative kidney/liver weight):

(1) $$\begin{array}{c} Relative\;\;weight=\frac{tissue\;\;weight\left(g\right)}{total\;\;body\;\;weight\left(g\right)}\times 100. \end{array}$$

### 2.7. Analysis of Hepatic and Kidney Function Parameters and Glucose Concentration

Plasma glucose, serum albumin, total protein, globulin, creatinine, and hepatic enzymes-aspartate transaminase (AST), alanine transaminase (ALT), and alkaline phosphatase (ALP) tests were measured using the automated RANDOX chemistry analyser. All procedures were followed according to the RANDOX kits manufacturer's guidelines.

### 2.8. Oxygen Radical Absorbance Capacity (ORAC)

ORAC is used to kinetically measure the ability of antioxidants in liver and kidney sample to scavenge radicals according to the method described by Ayepola et al. [24]. Fluorescence readings were measured by a Fluoroskan ascent plate reader (Thermo Fisher, Waltham, MA, USA) at 485 nm excitation and 538 nm emission wavelengths. The Fluoroskan took reading at the end of every 60 seconds for 2 hours. The scavenging effects of antioxidants were calculated by comparing the areas under the fluorescence curves of samples against the areas under curves of the controls. The regression equation (*Y* = *a* + *bX* + *cX* ^2^) was used to determine the ORAC values, where *Y* is trolox concentration in *μ*M and *X* is net area under the fluorescence decay curve. Results were reported in units called trolox equivalents (TE) per millilitre/*μ*mol.

### 2.9. The Ferric Reducing Antioxidant Power (FRAP)

Ferric reducing antioxidant power of tissue samples was assessed using the method that was developed by Benzi and Strain in 1996. FRAP is a colorimetric method that measures the ability of antioxidants to reduce oxidants. The method utilises Fe^3+^ ions as oxidants; the reducing power of the sample is determined by the conversion (reduction) of the ferric ion to the ferrous ion (Fe^2+^). Antioxidants donate electrons to the ferric ions thereby reducing them; however this method does not depend on the concentration of antioxidants. The reaction occurs at low pH where Fe^3+^ found in the (TPTZ) complex is reduced by antioxidants in a sample to the ferrous form (tripyridyltriazine complex) which is indicated by blue coloration. Changes in absorbance are determined using a spectrophotometer at a wavelength of 593 nm.

To determine the ferric reducing antioxidant power of tissue samples, the FRAP reagent was prepared by combining 30 mL of (300 Mm) acetate buffer at a pH of 3.6, 3 mL of TPTZ solution, 3 mL of FeCl_3_ solution, and 6 mL of distilled water. L-Ascorbic acid solution (1 Mm solution, 0.088 g of ascorbic acid + 50 mL distilled water) was used as the standard and used as a stock solution to prepare different concentrations of standards. 10 *μ*L of sample/standard was pipetted into the 96-well plates; each sample was triplicated. 300 *μ*L of the FRAP reagent was added giving a total volume of 310 *μ*L in each well. The mixture was incubated at 37°C for 30 minutes. After the incubation period, readings were taken and results were compared to a standard curve that uses an equation (*y* = *a* + *bx*). Results were expressed as *μ*mol AAEg^−1^ for both tissues.

### 2.10. Measurement of Endogenous Antioxidant Enzyme Activities

The antioxidant enzyme activities were evaluated in clear 96-well plate using the Multiskan plate reader (Thermo Fisher Scientific, USA). The catalase (CAT) activity was determined spectrophotometrically in tissue homogenates according to the modified method of Ellerby and Bredesen [25]. Superoxide dismutase (SOD) activity was assessed by the use of the modified method of Ellerby and Bredesen [25]. The reduced total glutathione (GSHt) concentration was determined by the method of Asensi et al. [26]. The Thermo Scientific Pierce BCA protein assay kit was used for the colorimetric detection and quantitation of protein.

### 2.11. Statistical Analysis

The data were expressed as mean ± standard error of the mean (SEM) and SD (standard deviation). Significant differences were analysed using the one-way analysis of variance (ANOVA). The Bonferroni multiple comparison analysis was used to compare differences among groups by the use of GraphPad*™* PRISM5 software. At the level of *P* value < 0.05 the differences were considered to be significant.

## 3. Results

### 3.1. *Hypoxis hemerocallidea* Effect on Serum Glucose Levels in Normal and STZ-Induced Diabetic Rats

Table 1 demonstrates the effect of* Hypoxis hemerocallidea* (200 mg/kg and 800 mg/kg) on glucose levels in both normal and STZ-induced diabetic rats. Results showed a significant reduction in glucose levels between the readings after STZ induction and the final glucose readings of group 3 following the administration of the aqueous extract of* H. hemerocallidea* (from 25.27 ± 2.282 mmol/L to 6.74 ± 2.452 mmol/L) and group 4 (from 24.53 ± 2.502 mmol/L to 10.17 ± 0.456 mmol/L) at *P* < 0.05. The greatest change was observed in group 3 and group 4 rats which had 73.3% and 58.54% change.

Table 2 illustrates the effect of* Hypoxis hemerocallidea* on total body weight and on relative kidney and liver weights. Induction of diabetes in groups 2, 3, and 4 significantly reduced the total body weights of rats when compared to the normal control group 1 and group 5 rats. Treatment of STZ-induced diabetic rats (group 3 and group 4) with* Hypoxis hemerocallidea* extract did not significantly increase the body weights when compared to the normal untreated control group 1 at *P* > 0.05 (229.2 g ± 44.90 g versus 303.75 g ± 23.68 g and 231.2 g ± 35.59 g versus 303.75 g ± 23.68 g), respectively. Injection of STZ significantly increased the relative kidney weights of group 2, group 3, and group 4 when compared to group 1 and group 5 animals indicating that treatment with* H. hemerocallidea* did not prevent kidney hypertrophy. Elevated relative liver weights were observed in groups 2 and 3 compared to the normal control group 2 and group 5. Treatment with 800 mg/kg of (aq)* Hypoxis hemerocallidea* failed to inhibit liver hypertrophy in STZ-induced diabetic group 3. Interestingly, treatment with 200 mg/kg of (aq)* H. hemerocallidea* significantly prevented liver hypertrophy in group 4 animals.

### 3.2. The Effects of* Hypoxis hemerocallidea* on Hepatic Biomarkers

Table 3 represents the result of* Hypoxis hemerocallidea* (aq) extract on hepatic biomarkers. The hepatic enzyme levels of serum in the diabetic control were significantly elevated when compared to the normal control group 1 and group 5. Daily dosage of 800 mg/kg of* Hypoxis hemerocallidea* failed to normalise the enzyme activities as indicated in Table 3. However, treatment with 200 mg/kg of* Hypoxis hemerocallidea* extract significantly reduced the serum levels of ALT and ALP in the diabetic group. Diabetic induction by STZ injection in groups 2, 3, and 4 significantly reduced serum levels of total protein, albumin, and globulin. Both dosages of* Hypoxis hemerocallidea* did not significantly reverse their serum levels.

### 3.3. Effects of* Hypoxis hemerocallidea* on Liver and Kidney Antioxidant Status

**Figure 2:**
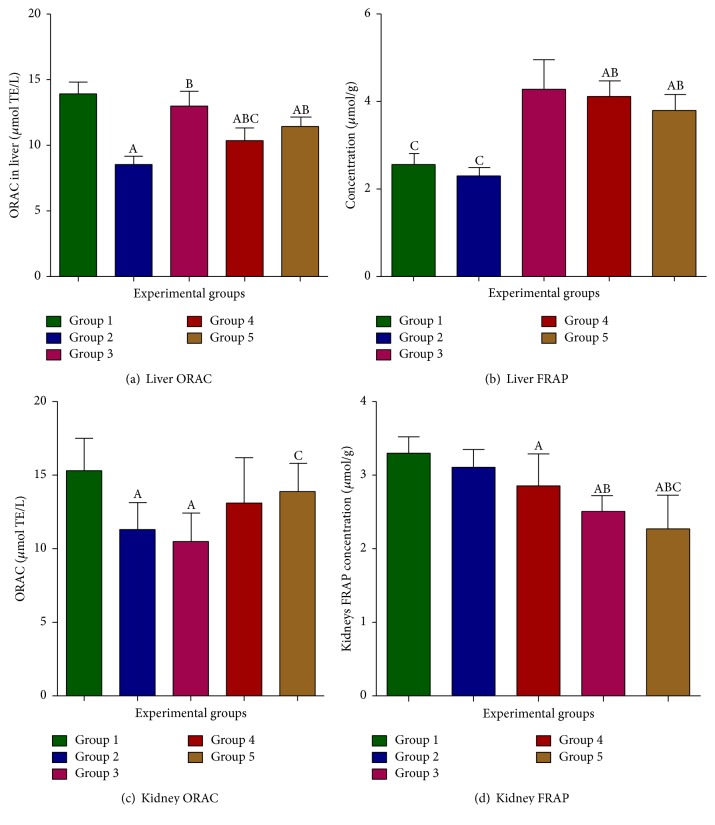
Effects of* H. hemerocallidea* on tissue antioxidant status. Group 1: normal control; group 2: diabetic control; group 3: diabetic group fed with 800 mg/kg of* H. hemerocallidea*; group 4: diabetic group fed with 200 mg/kg of* H. hemerocallidea*; group 5: normal controls (nondiabetic) fed with 800 mg/kg of* H. hemerocallidea*; A represents a significant difference when compared to group 1 control at *P* < 0.05; B indicates significant difference of groups when compared to group 2 at *P* < 0.05; and C represents significant differences of values in groups when compared to group 3; value in columns indicates means and ± standard error means (SEM).

Figures 2(a) and 2(b) graphs show the effect of* Hypoxis hemerocallidea* on liver oxygen radical antioxidant capacity (ORAC) and ferric reducing antioxidant power (FRAP). ORAC levels were not significantly altered in diabetic treated groups 3 and 4 when compared to the diabetic untreated group. As shown in Figure 2(c), the ORAC was reduced in the kidneys of diabetic control rats compared to nondiabetic control group 1 and group 5 rats. Induction of diabetes with STZ resulted in elevation of kidney ferric reducing antioxidant power in the diabetic control group with no significant differences noted when compared to the normal control group 1 and group 3. FRAP levels in groups 4 and 5 rats were significantly lower when compared to the normal control group 1 and the diabetic untreated group (Figure 2(d)).

### 3.4. The Effects of* Hypoxis hemerocallidea* on the Activities of Endogenous Antioxidant Enzymes in Liver and Kidney Tissues

Table 4 represents the effects of* Hypoxis hemerocallidea* on endogenous antioxidant enzymes in experimental rats. Although no change was observed in liver SOD activities among all groups, liver CAT activities improved following* Hypoxis hemerocallidea* treatment in diabetic groups.* Hypoxis hemerocallidea* administration in groups 3 and 4 showed no significant effect on hepatic GSHt concentration when compared to the normal and diabetic controls. In the kidney tissue, CAT activity in the diabetic control was increased significantly when compared to groups 1, 4, and 5. Treatment of diabetic groups with the 2 dosages of* Hypoxis hemerocallidea* in comparison to the diabetic control did not significantly improve altered kidney SOD activity and GSHt concentration. The control group 5 treated with 800 mg/kg of* H. hemerocallidea* showed increased SOD activity and GSHt concentration when compared to all the groups.

## 4. Discussion

Medicinal plants possess potent therapeutic metabolites that have been linked to the medicinal values or medicinal activities of plants [27]. These metabolites include but not limited to the following: carotenoids, flavonoids, alkaloids, polyphenols, terpenoids, sterols, and glucosides [27]. These compounds work synergistically in hyperglycemic states to exert hypoglycemia. It has been reported that hypoglycemia is achieved by these compounds' abilities to reverse or reduce insulin resistance, increase the hepatic glucose output, decrease the rate of digestion, and stimulate insulin output and inhibition of intestinal glucose absorption [28].* Hypoxis hemerocallidea* corm is rich in phytosterols (*β*-sitosterol) and hypoxide (that is converted to rooperol in the gut). These two compounds have been speculated to be responsible for its hypoglycaemic and antioxidant effects [29].

In diabetes mellitus, proper monitoring and regulation of blood glucose levels have been linked to delayed onset of DM complications [30]. Induction of diabetes using STZ in rodents results in the destruction and necrosis of the beta cells of the pancreas which consequently leads to diminished insulin release and elevated blood glucose levels [31]. In this present study, significant elevation in the fasting blood glucose levels up to the diabetic range (groups 2, 3, and 4) was observed 48 hours after STZ-induced diabetes. Following treatment with* Hypoxis hemerocallidea* (aq) extract for 6 weeks, there was a significant decrease in fasting glucose levels in the diabetic treated groups 3 and 4. The greatest percentage change of 73.3% in the fasting glucose levels was observed in the STZ-induced diabetes group treated with 800 mg/kg of the* Hypoxis hemerocallidea* (aq) extract followed by the group treated with a lower dose of 200 mg/kg* Hypoxis hemerocallidea* (aq) extract with a 58.54% decrease. The glucose lowering effect observed in this study correlated with the results obtained by Ojewole [20] in an acute study where he reported reduced fasting glucose levels in diabetic rats using the same plant. Absence of glucose modification observed in group 5 (normal treated group) in this study is in contrast to proposition that consumption of* Hypoxis hemerocallidea* (aq) extract in healthy subjects causes hypoglycemia. The results of the effect of the* Hypoxis hemerocallidea* (aq) extract in normal rats disagree with the results obtained by Ojewole [20] where he reported a decrease in fasting blood glucose levels in normal rats. The mechanism of blood glucose level reduction is still obscure; however, it is suggested that the plant exerts a short lived antihyperglycemic effect in which the mechanism mimics that of metformin [32].

Diabetes mellitus is associated with rapid weight loss as a consequence of uncontrolled catabolism of structural proteins as a compensatory response against abnormal carbohydrate metabolism. Muscle atrophy in diabetic subjects is due to a combination of decreased protein synthesis, increased gluconeogenesis, and increased protein degradation [33]. Administration of the extract of* Hypoxis hemerocallidea* (aq) in diabetic treated group did not significantly alter body weights despite the plant's glucose lowering effect (antihyperglycemic).

Diabetic nephropathy (DN) is labelled as a major complication of diabetes mellitus that affects about 40% of all diabetic patients. DN is characterised by both structural and functional features which include proteinuria, reduced glomerular filtration rate, renal hypertrophy, increased blood pressure, and decreased creatinine clearance [34]. The emergence of renal dysfunction in this study was confirmed by increased relative kidney weights, an early event in the progression of glomerular pathology observed in the diabetic control (group 2) and the diabetic group treated with 800 mg/kg* Hypoxis hemerocallidea* extract (group 3). Results from this study suggests that the increase in kidney weights in diabetic rats may have been caused by hyper filtration, aggregation of lymphocyte and fat infiltrations, and glomerular hypertrophy which indicates increase in size and area of the glomeruli. Our findings in abnormal kidney function correlated with results of Musabayane et al. [32] who reported that* Hypoxis hemerocallidea* reduced the glomerular filtration rate which implies the possible accumulation of toxic substances, for example, urea and increase in serum creatinine levels. Elevated concentration of serum creatinine was seen in the diabetic control when compared to the normal control and group 5 indicating abnormal creatinine clearance by the kidneys and thus further confirming kidney dysfunction that can also be linked to decreased GFR [32].

The pathological effects of hyperglycemia and insulin resistance on the hepatic tissue have been demonstrated by elevated serum hepatic enzymes and liver hypertrophy. In this study, increased relative liver weights in the diabetic control and groups 3 and 4 may be due to hypoinsulinemia following STZ-induced diabetes. Hypoinsulinemia triggers lypolysis resulting in excess free fatty acids (FFAs) accumulations and storage in the hepatic tissue resulting in hypertrophy [35].* Hypoxis hemerocallidea* (aq) extract did not significantly ameliorate liver hypertrophy in the STZ-induced diabetes treated groups. The AST, ALT, and ALP are hepatic enzymes that are assessed to monitor hepatic integrity and cardiac injuries. Elevation of serum liver enzymes is linked to hepatic and cardiac pathologies which are complications of diabetes. On the other hand, ALT is found predominantly in the hepatocytes and therefore increased activities of both AST and ALT in the serum strongly point to hepatic injury [36].

In previous literature, increased serum activity of ALP in hyperglycemic environment was linked to the peroxidation of lipids in the cell membranes of hepatic cells [37] whereas leakage of AST and ALT from the hepatocytes' cytosol into the blood was reported to be caused by the disruption of hepatocytes' cell membranes due to accumulation of toxic free fatty acids (FFAs) [35]. In this current study, there was a marked increase in serum activities of hepatic enzymes in group 2 (increase in all hepatic enzymes), group 3 (increase in AST and ALP), and group 4 (increase in ALP) when compared to the normal control group. The increase in serum hepatic enzymes in the STZ-induced diabetic groups (2, 3, and 4) can be explained not only by the hyperglycemia mediated hepatic injury but possibly by the hepatotoxic effects of STZ itself since the liver is the organ responsible for drug metabolism. This implies that abnormal liver functions are as a result of combined hepatotoxic effects of STZ and excess glucose levels.

Decreased levels of both albumin and total protein in diabetic patients may occur consequent to decreased protein synthesis rate. Protein synthesis in diabetics is derailed because the mechanisms involved in protein synthesis require ATP from glucose metabolism, in which glucose metabolism is attenuated in diabetes. Gluconeogenesis or protein catabolism occurs in diabetes in an attempt to balance ATP production [38]. In the present study, there were significant decreases in the serum levels of total protein and albumin in groups 2, 3, and 4 when compared to the normal control and group 5. Reduced levels of the proteins may be due to hyperglycemia which targets proteins that would then be utilised in advanced glycation end products (AGEs) formation. Other reasons to explain the decrease involves increase in protein catabolism, decreased synthesis of proteins, liver damage, and renal loss (polyuria) due to renal impairment.

Oxidative stress has been documented as a critical participant in the pathogenesis of various diseases as it causes damage to cellular components resulting in loss of cellular function [39]. During oxidative stress conditions, the sources of endogenous antioxidants are restricted and hence incorporation of exogenous antioxidants is essential to replenish the total antioxidant pool. It is our opinion that* Hypoxis hemerocallidea* has the potential to contribute to the antioxidant pool of a biological system as shown in this study.

In diabetes mellitus, the reactive oxygen species scavenging power of antioxidants becomes weakened resulting in diabetes induced oxidative stress [27]. In our study, the total antioxidant capacity in both liver and kidney tissues was assessed by the ORAC method. The ORAC is an oxidation inhibition based method that measures the ability of a molecule or specimen to inhibit the oxidation of a fluorescent probe derived from peroxyl radicals. There was no significant difference in liver ORAC levels of group 3 and group 5 when both were compared to the normal control group 1, which suggests the antioxidant power of the* Hypoxis hemerocallidea* at a dose of 800 mg/kg. The antioxidant capacity in the diabetic group treated with 200 mg/kg was increased with no significant differences to group 1 and group 5. Therefore, it is possible to suggest that the 200 mg/kg dosage of* Hypoxis hemerocallidea *induces desirable antioxidant effects compared to the 800 mg/kg dosage in the kidneys.

The ferric reducing antioxidant power assay (FRAP) was also performed to assess and confirm the presence of functional recovery in the treated groups after induction of diabetes with STZ. There was increased ferric reducing antioxidant power in the liver of the treated groups 3, 4, and 5 when compared to untreated group 1 normal control and the diabetic control (group 2). It appears that at both dosages of the plant in groups 3 and 4 the reducing antioxidant power of the plant was rejuvenated when compared to the diabetic control group. The seemingly decreased levels of FRAP in the normal control group (group 1) could be due to the nonpathological state, hence no extra demand for antioxidants to curtail less amounts of ROS. The unexpected increase in FRAP in the diabetic control could be due to chronic hyperglycemia that occurred after the induction of diabetes with STZ. Increase in FRAP in diabetic rats was linked to ketosis [40] in which the authors reported that oxidative metabolism is dependent on time period. In addition, it also reported that, during acute hyperglycemia, the FRAP levels tend to decrease while it later increases during chronic hyperglycemic phases which could be the case in our study as increase in enzyme activities indicates increase in demand to meet up with oxidative stress requirement [27].

Catalase (CAT) is endogenous antioxidant that highly determines hepatic antioxidant status. It converts H_2_O_2_ to water and oxygen and renders the harmful peroxides inactive. The hepatic catalase results of our study showed a significant decrease in the catalase activity of group 2 diabetic rats when compared to all the groups. These results correlated with that of Ayeleso et al. [41] who reported a significant decrease in hepatic catalase activity in diabetic rats. The decrease in catalase activity in the diabetic hepatic tissue signifies the failure of catalase to detoxify hydrogen peroxide resulting in increased oxidative damage. No clear changes were noted in diabetic treated groups 3 and 4 when compared to the normal control and in group 5, thus indicating the ameliorative and upregulatory effects of the plant extract on catalase activity. In diabetic treated groups, the extract of* Hypoxis hemerocallidea* increased catalase suggesting the plant's potential antioxidant power. The catalase activity in the diabetic kidney tissue (group 2) was increased when compared to group 4 and group 5. The increase in catalase activity in the diabetic group was in line with the results of Qujeq and Rezvani [42] who argued that the increase in catalase activity in diabetic rats was due to the uncontrolled ROS production as a consequence of hyperglycemia which upregulated the expression of catalase.

SOD enzyme catalyses the dismutation of the superoxide anion to hydrogen peroxide and oxygen. Superoxide dismutases are mainly found in the cytosol attached to metal ions such as copper, zinc, and manganese. The products of superoxide dismutation are further catalysed by catalase into water and oxygen. It has been reported that hyperglycemia causes significant decreases in SOD activities in the tissues of diabetic rats as a result of inactivation of SOD by the hydrogen peroxide or by glycation [43]. We did not observe significant difference in the SOD activities in the liver tissue of all groups when compared to the normal control group 1. Dias et al. [44] reported that the discrepancies encountered when assessing antioxidant enzyme activities were due to differences in tissue specificity, variation in disease severity, and duration of the disease. In the kidney tissue, the normal treated group 5 showed significant increased SOD activity when compared to the activities of all groups, which suggest the ability of* H. hemerocallidea* to boost renal SOD activities thereby improving the antioxidative status when compared to the normal untreated group 1.

GSH is another important endogenous antioxidant and a cofactor of some enzymes taking part in the reduction of ROS; hence it has been regarded as a marker of free radical damage [26]. The hepatic GSHt concentration in diabetic controls was significantly reduced when compared to group 1 and group 5. Decrease in the hepatic GSHt level was linked to the depletion of GSHt stores possibly encountered during free radical scavenging in response to hyperglycemia induced oxidative stress. Treatment of the diabetic treated group 3 and group 4 rats with* Hypoxis hemerocallidea* (aq) extract showed a nonsignificant increase in the GSHt concentration compared to the diabetic control group. A significant elevation in GSHt concentration was observed in the kidney tissue of the normal treated group (group 5) when compared to all the groups.

## 5. Conclusion

Based on the finding of this study,* Hypoxis hemerocallidea* demonstrated enhanced antioxidant activity and antihyperglycemic effect in the group that experienced greater glucose reduction. However, at higher concentration, a negative effect on the kidneys was observed; therefore further study is recommended.

## Figures and Tables

**Table 1:** The effect of *H. hemerocallidea* on glucose concentrations.

Groups	Glucose reading after STZ induction (mmol/L)	Final glucose reading after 6 weeks (mmol/L)	Percentage change (%)
Group 1	6.23	5.31	1.47
Group 2	29.54	24.73	1.62
Group 3	25.27	6.74	73.3
Group 4	24.53	10.17	58.54
Group 5	6.25	5.07	1.89

Group 1: normal control; group 2: diabetic control; group 3: diabetic group fed with 800 mg/kg of *H. hemerocallidea*; group 4: diabetic group fed with 200 mg/kg of *H. hemerocallidea*; group 5: normal controls (nondiabetic) fed with 800 mg/kg of *H. hemerocallidea*.

**Table 2:** Effect of *H. hemerocallidea* on total body weight and relative weights of kidney and liver tissues.

	Group 1	Group 2	Group 3	Group 4	Group 5
Total body weight (g)	303.8 ± 23.7	214.7 ± 25.5^ae^	229.2 ± 44.9^ae^	231.2 ± 35.6^ae^	302.9 ± 25.1
Kidney weight (g)	1.99 ± 0.05	2.51 ± 0.09	2.34 ± 0.09	2.22 ± 0.09	1.93 ± 0.05
Relative kidney weight (g)	0.66 ± 0.02	1.17 ± 0.05^a^	1.02 ± 0.01^a^	0.96 ± 0.02^a^	0.63 ± 0.01^bcd^
Liver weight (g)	10.47 ± 0.41	10.15 ± 0.42	9.89 ± 0.50	9.11 ± 0.33	10.13 ± 0.29
Relative liver weight (g/100 g)	3.45 ± 0.01	4.72 ± 0.02^a^	4.31 ± 0.05^a^	3.94 ± 0.02	3.34 ± 0.01^bc^

Group 1: normal control; group 2: diabetic control; group 3: diabetic group fed with 800 mg/kg of *H. hemerocallidea*; group 4: diabetic group fed with 200 mg/kg of *H. hemerocallidea*; group 5: normal controls (nondiabetic) fed with 800 mg/kg of *H. hemerocallidea*; a represents a significant difference when compared to group 1 control at *P* < 0.05; b indicates significant difference of groups when compared to group 2 at *P* < 0.05; c represents a significant differences of values in groups when compared to group 3; d indicates significant difference of groups when compared to group 4 at *P* < 0.05; and e indicates significant difference when compared with control group 5 *P* < 0.05.

**Table 3:** Effects of *H. hemerocallidea *on serum levels of liver enzymes, total protein, and albumin.

	Group 1	Group 2	Group 3	Group 4	Group 5
AST (U/L)	142.20 ± 11.3	235.5 ± 39.5^a^	237.7 ± 26.4^a^	183.6 ± 20	179.5 ± 15.9^c^
ALT (U/L)	74.22 ± 7.87^b^	120.9 ± 8.56	85.7 ± 8.07^b^	79.8 ± 11.6^b^	79.3 ± 6.84^b^
ALP (U/L)	136.5 ± 8.47	537.9 ± 68.5^a^	424.2 ± 71.3^a^	214.9 ± 50.9^abc^	134.9 ± 11^bc^
Total protein (g/L)	56.5 ± 0.79	50.59 ± 1.87^a^	50.31 ± 0.29^a^	51.2 ± 0.8280^a^	54.4 ± 0.73^bc^
Albumin (g/L)	33.19 ± 0.33	30.01 ± 0.34^a^	20.9 ± 0.54^a^	30.28 ± 1.24^a^	32.6 ± 1.29^bcd^

Group 1: normal control; group 2: diabetic control; group 3: diabetic group fed with 800 mg/kg of *H. hemerocallidea*; group 4: diabetic group fed with 200 mg/kg of *H. hemerocallidea*; group 5: normal controls (nondiabetic) fed with 800 mg/kg of *H. hemerocallidea*; a represents a significant difference when compared to group 1 control at *P* < 0.05; b indicates significant difference of groups when compared to group 2 at *P* < 0.05; c represents a significant differences of values in groups when compared to group 3; and d represents significant difference of groups when compared to group 4; value in columns indicates means and ± standard error means (SEM).

**Table 4:** Effects of *H. hemerocallidea* on activities of endogenous antioxidant enzymes in liver and kidney tissue.

	Group 1	Group 2	Group 3	Group 4	Group 5
Liver					
CAT (*μ*mol/min/*μ*g)	50.52 ± 1.61	39.41 ± 1.18^a^	50.74 ± 3.0^b^	52.02 ± 2.63^b^	52.8 ± 1.84^b^
SOD (U/*μ*g)	41.46 ± 0.68	39.34 ± 0.45	41.84 ± 0.51	40.37 ± 0.6	41.54 ± 0.62
GSHt (*μ*mol/g)	5.02 ± 0.31	3.06 ± 0.44^ae^	4.64 ± 0.56	4.17 ± 0.38	5.39 ± 0.3
Kidney					
CAT (*μ*mol/min/*μ*g)	28.64 ± 2.27	35.58 ± 0.8^a^	31.46 ± 1.49	26.38 ± 1.34^b^	24.79 ± 1.25^bc^
SOD (U/*μ*g)	43.58 ± 1.29	40.96 ± 1.17	42.50 ± 0.67	43.55 ± 0.45	48.18 ± 0.57^abcd^
GSHt (*μ*mol/g)	6.33 ± 0.19	5.10 ± 0.28	6.10 ± 0.18	6.42 ± 0.26	7.92 ± 0.51^abcd^

Group 1: normal control; group 2: diabetic control; group 3: diabetic group fed with 800 mg/kg of *H. hemerocallidea*; group 4: diabetic group fed with 200 mg/kg of *H. hemerocallidea*; group 5: normal controls (nondiabetic) fed with 800 mg/kg of *H. hemerocallidea*; a represents a significant difference when compared to group 1 control at *P* < 0.05; b indicates significant difference of groups when compared to group 2 at *P* < 0.05; c represents a significant differences of values in groups when compared to group 3; d represents significant difference of groups when compared to group 4; and e represents the significant difference of groups when compared to group 5; value in columns indicates means and ± standard error means (SEM).
